# Ankle distraction arthroplasty for the treatment of severe ankle arthritis: Case report, technical note, and literature review

**DOI:** 10.1097/MD.0000000000022330

**Published:** 2020-09-25

**Authors:** Xiao-Ning Liu, Fei Chang, Han-Yang Zhang, Zhuan Zhong, Pan Xue, Bing-Zhe Huang

**Affiliations:** Orthopaedic Medical Center, The Second Hospital of Jilin University, Changchun, Jilin Province, China.

**Keywords:** ankle arthritis, ankle distraction arthroplasty, arthrodesis, pain

## Abstract

**Rationale:**

Widely applied in the treatment of severe ankle arthritis (AA), ankle distraction arthroplasty (ADA) can avoid not only the ankle range of motion loss but also ankle fusion. However, the clinical outcomes of ADA for severe AA are poorly understood. This study aims to present our clinical outcomes of severe AA treated by ADA.

**Patient concerns:**

A 53-year-old man suffered right ankle sprain 10 years ago, endured right ankle pain and limited movement for 6 years.

**Diagnosis:**

The patient was diagnosed as severe AA.

**Interventions:**

He received ankle distraction arthroplasty. No adjuvant procedures were performed. The visual analog scale (VAS), the American Orthopaedic Foot and Ankle Society (AOFAS) score, the short-form (SF)-36 physical component summary (PCS) score and ankle activity score (AAS) were recorded to access the clinical outcomes pre- and postoperatively. Moreover, ankle joint space distance was evaluated on weight-bearing radiographs.

**Outcomes:**

The patient derived effective pain relief and restored a satisfactory range of movement. There was a 13-month follow-up period after frame removal. The AOFAS score improved from 56 preoperatively to 71 postoperatively. The VAS score decreased from 6 prior to surgery to 1 after surgery. The SF-36 PCS was 47.2 and 71.8 pre- and postoperative, respectively. The AAS scores were improved from 3.4 preoperatively to 7.3 postoperatively.

**Lessons:**

ADA is reliable to achieve pain relief, functional recovery, and serve AA resolution. Besides, it is an alternative to ankle arthrodesis or total ankle arthroplasty in selected patients with severe AA.

## 1 Introduction

Ankle arthritis (AA) is a progressive disease caused by trauma, characterized by weight-bearing pain, spontaneous pain and limited function, which usually has a great impact on life and work.^[1–6]^

According to previous studies, the traditional methods to treat advanced-stage AA require the internal fixation of ankle joint, such as tibial-talus arthrodesis, total ankle arthrodesis.^[7–11]^ However, these methods may affect adjacent joints and lead to arthritis of adjacent joints, especially in case of malunion after fusion.^[12,13]^ To preserve ankle motion and avoid the occurrence of adjacent arthritis, scholars have developed several strategies for the treatment of AA, including total ankle arthroplasty,^[10,13,14]^ ankle joint debridement,^[3]^ treatment of cartilage and bone marrow microfracture,^[2,4,15]^ autogenous cartilage transplantation, and allogenic cartilage transplantation. However, the long-term results remain less than satisfactory.

In recent years, ankle distraction arthroplasty (ADA) was identified as a promising method to treat AA. However, the clinical outcomes of ADA for severe AA are poorly understood. Therefore, the purpose of this paper is to present the clinical findings of our study, as well as to review the origin, mechanism, devices type, technique, advantages, prognostic indicators, and complications of ADA.

### 1.1 Ethics

This case report was approved by the institutional review board of the second hospital of Jilin University. Informed written consent was obtained from the patient for publication of this case report and accompanying images.

## 2 Case report

### 2.1 Patient characteristics

A 53-year-old man presented himself to the Foot and Ankle Department of the Second Hospital of Jilin University. His major symptoms included right ankle pain and limited movement that had been persistent for 6 years. He had a 10-year history of right ankle sprain. The patient was only capable of limited movement due to the persistent pain in his ankle.

**Figure 1:**
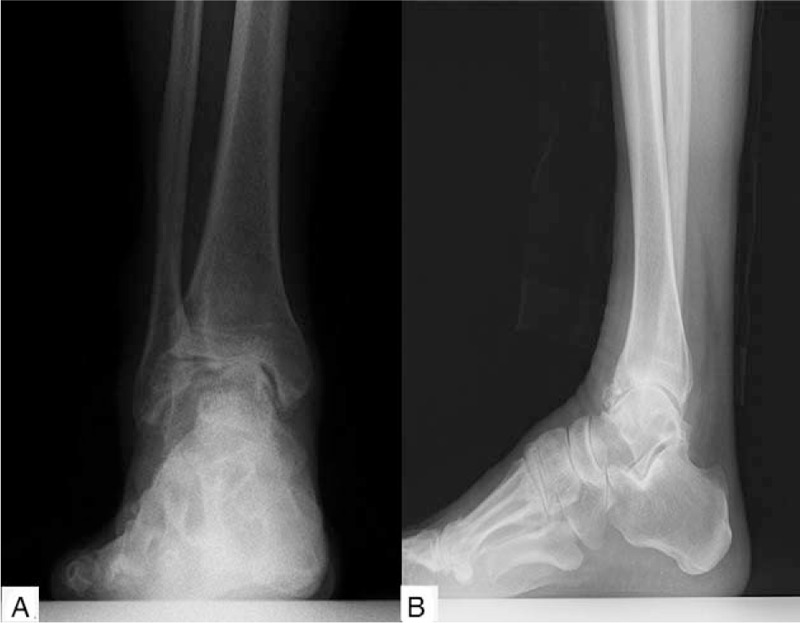
The weight-bearing positive (A) and lateral (B) radiograph of right ankle revealed many osteophytes and narrow joint space, especially in the medial side.

**Figure 2:**
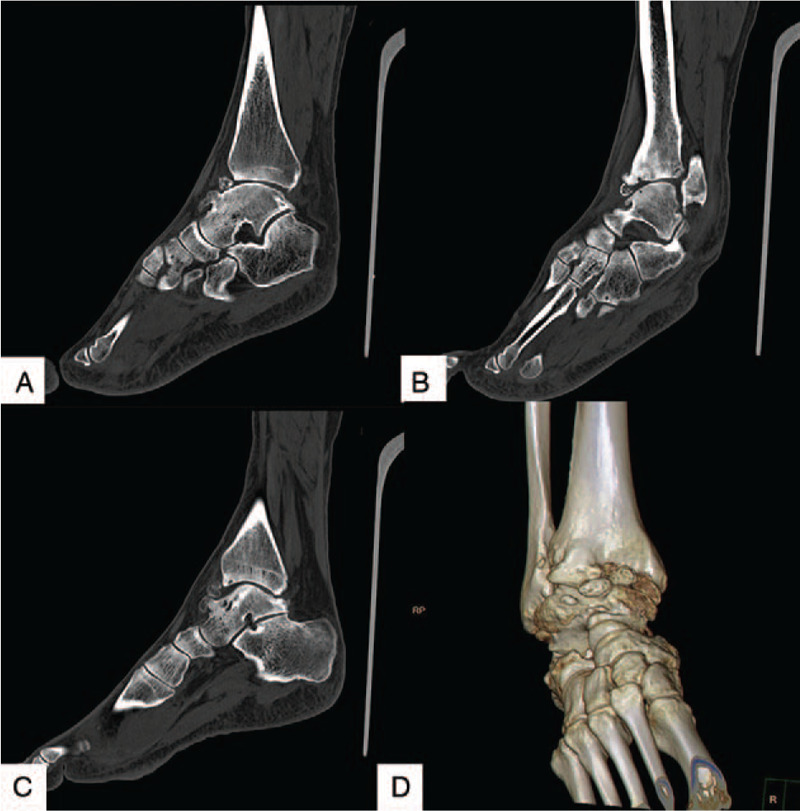
CT scan (A to C) showed sclerotic borders under cartilage and cystic changes in tibia and talus. It also revealed narrowing joint space and osteophytes on the talus and around the ankle joint (D).

As revealed by the weight-bearing anteroposterior and lateral radiograph of the right ankle, the joint space narrowed, especially in the medial side, and there were plenty of osteophytes around the joint (Fig. 1). A three-dimensional computed tomography (Fig. 2) showed sclerotic borders under cartilage and cystic changes in tibia and talus. It also revealed narrowing joint space and osteophytes both on the talus and around the ankle joint.

The conservative approaches to treatment include restricted activity, oral non-steroidal anti-inflammatory drugs, bracing, physical therapy, and steroid injection therapy, all of which were trialled but failed. Under this circumstance, the patient received ADA.

The visual analog scale (VAS), American Orthopaedic Foot and Ankle Society (AOFAS) score, the short-form (SF)-36 physical component summary (PCS) score, and ankle activity score (AAS) were recorded to access the clinical outcomes pre- and postoperatively. Ankle joint space distance was evaluated as well by weight-bearing radiograph plains and CT.

### 2.2 Surgical technique

After general anesthesia, the electric tourniquet was tied to the patient's thigh. The operative approach involved midline incision, which was performed between the tibialis anterior and extensor hallucis longus tendons and was approximately 7.0 cm in length. The capsular was opened after initial exposure, and the ankle was revealed. Several loose bodies were discovered and then removed. Osteophytes on the anterior, medial, and lateral aspects of the joint were resected. However, impingement remained visible when the ankle was passively dorsiflexed, inversed, and reversed. Thus, osteophytes, along with the anterior, medial, and lateral aspects of the talus, were resected as well. The cartilage damage with a size of about 5 cm × 3.5 cm was found out in tibia and talus, and under the damaged cartilage sclerotic borders were discovered. A spatula was applied to trim the damaged areas, and a Kirschner wire with a diameter of 1.5 mm was used to drill at the site of cartilage damage until bleeding. Then, the incision was sutured layer by layer after repeated washing with physiological saline. A circular external fixator was mounted onto the right calf 5 cm clear of the ankle and fixed with 4 tension needles. Then an articulated fixator was mounted onto the right foot with its axial parallel to the ankle joint. Two tension needles were attached to the U-shaped ring in 2 different directions for the purpose of making the ring parallel to the plantar of the foot. A tension needle was also fixed to the U-shaped ring by the 5 metatarsals. As a result, a 7-mm distraction was applied across the joint intraoperatively.

### 2.3 Clinical outcomes and follow-up

**Figure 3:**
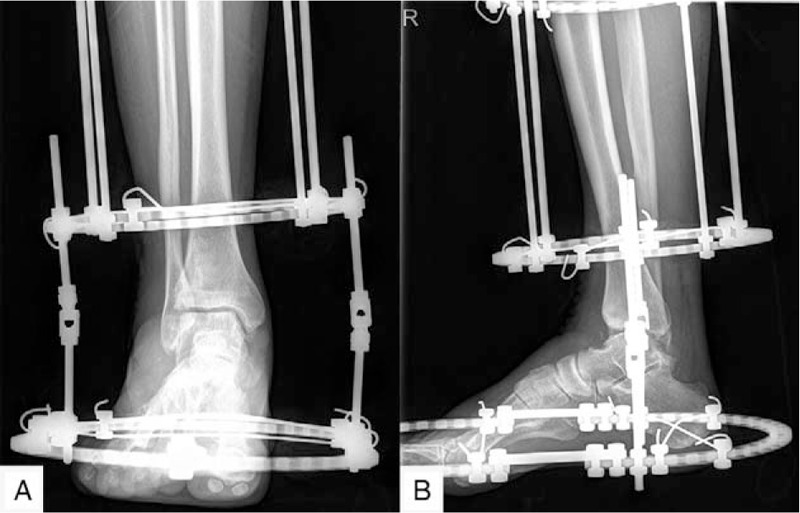
The radiograph 1st week postoperative showed good joint space and alignment.

**Figure 4:**
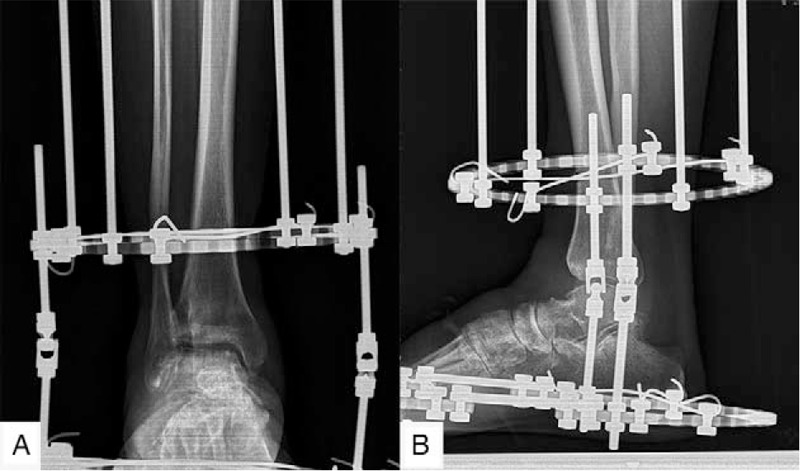
The weight-bearing radiograph 3rd month postoperative showed that the joint space remained good.

**Figure 5:**
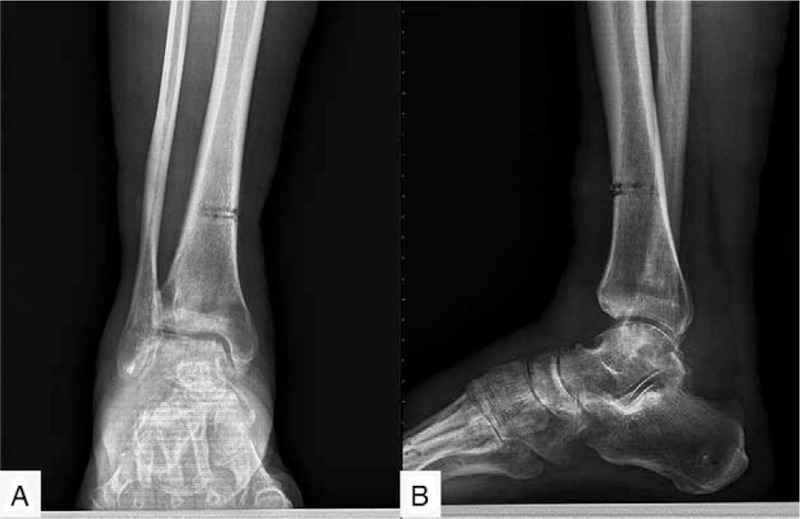
The weight-bearing radiograph showed that the joint space remained good after the external fixator was removed.

**Figure 6:**
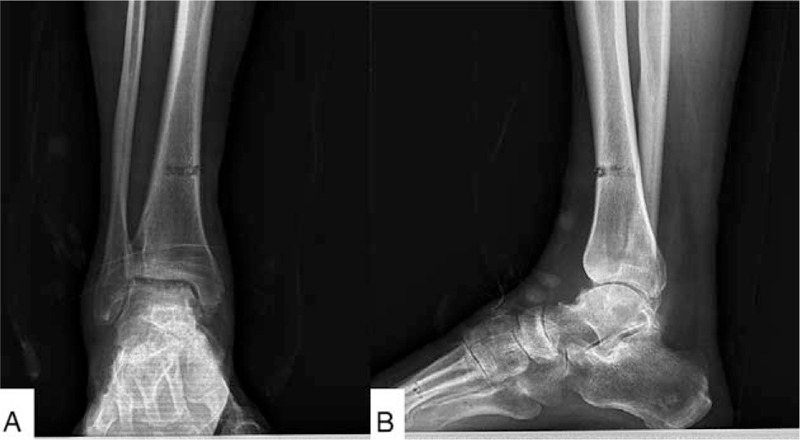
The weight-bearing radiograph showed that the joint space remained clear 3 mo after the external fixator was removed.

Intravenous antibiotics were prescribed to the patient within 24 hours after surgery. The patient took intravenous non-steroidal anti-inflammatory drugs (NSAIDs) in the first 3 days and NSAIDs orally for 1 week. The incision care was carried out every 2 or 3 days until the sutures were removed at the 14th day postoperatively. Pin care started from day 2 after surgery and involved the cleaning of pin sites with 75% alcohol. The patient came for follow-up at the 1st week. The radiographs showed functional joint space and alignment (Fig. 3). The next follow-up was scheduled for the 3rd month after the operation. The radiograph (Fig. 4) revealed that the joint space remained in good condition, so that the external fixator was removed in the operating room without anesthesia. The weight-bearing radiographs (Fig. 5) showed that the joint space remained in good condition postoperatively, so that the patient was asked to walk with the assistance of a walking cast after the operation. The weight-bearing radiograph showed that the joint space remained clear 3 months after the external fixator was removed (Fig. 6).

The AOFAS score was improved from 56 preoperatively to 71 postoperatively. The VAS score decreased from 6 prior to surgery to 1 after surgery. The SF-36 PCS was 47.2 and 71.8 pre- and postoperative, respectively. The AAS scores were improved from 3.4 preoperatively to 7.3 postoperatively. No complication occurred during 13 months of follow-up visit.

## 3 Discussion

AA is usually caused by the alteration to bone structure and ligament damage after trauma.^[4,7,16–19]^ Although suitable open treatment is beneficial to improve ankle function, it can still cause changes to biomechanics and anatomical structure.^[18]^ The initial goal for the management of AA is to reduce pain and restore ankle function as much as possible.^[20]^ Joint preservation surgical strategies for the management of AA mainly include lifestyle adjustment and oral non-steroidal anti-inflammatory drugs.^[3]^ However, it is only suitable for the early stage of AA and is ineffective for the treatment of advanced and severe AA. Meanwhile, joint sacrifice is mainly related to arthrodesis.^[7–11,21,22]^ However, arthrodesis carries such potential risks as continuous pain, declining function, and adjacent joint arthritis.^[7,8,11,23]^ Superior malleolus osteotomy is suitable for the early stage of ankle arthritis with partial reservation of ankle joint surface, frontal alignment deformity of ankle joint, and reservation of ankle joint motion.^[6,24–26]^ Therefore, it is of considerable significance to develop a method that can maintain the range of movement in the treatment of advanced AA. In this study, ADA was applied to treat serve AA and a satisfactory effect was achieved. The purpose of this study is to present our case and to review the characteristics of ADA (Table 1).

**Table 1:**
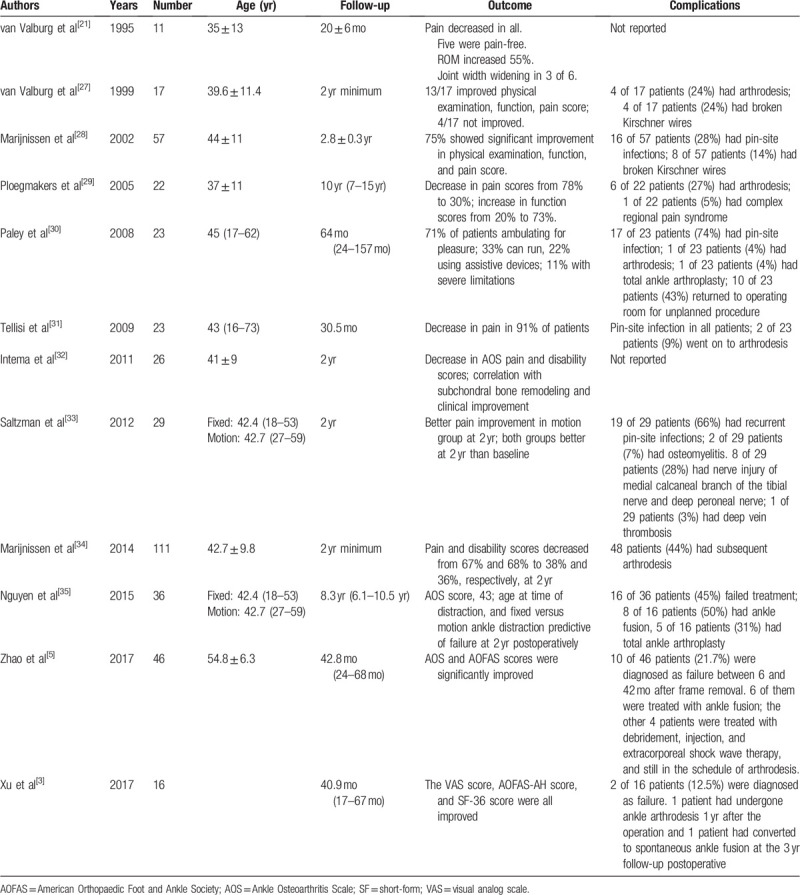
Literature review.

Concerning the origin of ADA technique, in 1978, Judet and Judet^[36]^ first reported a technique of mechanical opposite direction of the joint surface using an external fixator that allows “fibrous tissue between the bones ends” to find an alternative to total arthroplasty for arthritis. They first removed the joint cartilage from the end of the tibiotarsal of the dog, gave it 30-day and 4 to 8 mm distraction, and then conducted a histological analysis of the regenerated tissue. A year later, they found out that hyaline cartilage, similar to normal cartilage, was regenerated on the surface of the joint.^[36]^ In 1994, Aldegheri et al^[37]^ described 80 cases of hip arthritis treated with distraction therapy. After the good results of 46 patients who were followed for at least 5 years, it was concluded that the results of radiographic findings are not necessarily associated with the clinical outcomes. In 1995, van Valburg et al^[21]^ published a report on ADA, after which this technique became widely applied.

The mechanism of ADA remains unclear. Despite this, it was demonstrated by scholars that it is related to non-weight-bearing on the surface of the ankle joint after mechanical distraction, which is beneficial to cartilage regeneration.^[21,38–41]^ The rigidity of the circular ring fixator can provide enough stress shielding at the ankle joint and allow subchondral bone reconstruction, which has been proved to bring clinical benefits.^[32]^ However, its clinical relevance to pain relief is still controversial. In the present study, the pain was effectively controlled postoperatively. In our view, the pain caused by ankle arthritis is associated with the pressure of the fluid in the joint into the subchondral bone. Subsequent to the arthroplasty, the mechanical stress and irritation of the cartilage are reduced, thus alleviating the pain and promoting cartilage repair.

Regarding the devices type of ADA, it can be divided into fixed distraction and mobilizable distraction.^[3,31]^ Saltzman and his colleagues^[33]^ conducted a prospective randomized controlled trial, which led to the discovery that the clinical outcomes of mobilizable distraction were better compared to a fixed distraction. This view is also supported by Xu et al.^[3]^ On the contrary, Nguyen et al^[35]^ counterargued that mobilizable distraction was superior to fixed distraction due to the limited number of patients. However, the preoperative and postoperative distraction distance in their study was not referred to.

Meanwhile, a circular frame may be better than a mono-lateral fixator, which is because the latter conveys uneven distraction by the cantilever mechanics. Moreover, it is challenging to place the mono-lateral fixator with a simple hinge along the ankle axis.^[31]^ Therefore, in our study, a fixed frame with a circular shape was selected for ankle distraction and excellent clinical outcomes were achieved. The positive results are partially attributed to the proper selection of the distraction device.

As for the technique of ADA, in 1995, van Valburg et al^[21]^ established the clinical standard for ankle distraction, which is weight-bearing radiographs showing an ankle gap of 5 mm during 12 weeks of joint distraction. In 2017, Xu et al^[3]^ found out that the space of ankle joint distraction was reduced after the external fixator was removed. However, if the joint gap is greater or equal to the standard value within 1 year postoperatively, the joint gap may remain in this state for a long time. According to previous studies,^[21,27,29,42]^ it was found out that chondrocytes may require 3 to 6 months of non-weight-bearing state to rebuild the cartilage matrix, and the full clinical benefit was not received until many months after joint distraction. Therefore, it was speculated that time plays a significant role in achieving stable soft-tissue intervention.^[1,31]^ In the present case, ankle distraction distances of 7 mm were maintained for 4 months, and no reduction in ankle gap was observed by radiographic after the fixation frame was removed. In our view, after joint distraction, subchondral sclerosis of the ankle is effectively prevented as the axial load is transmitted through the external fixator rather than through the joint. Besides, vascular reconstruction is conducive to the repair of osteoarthritis.

With reference to the prognostic indicators of ADA, it includes the ankle space, Ankle Osteoarthritis Scale (AOS) score, age, and gender. Postoperatively, ankle space was validated as an important prognostic indicator by Tellisi et al.^[31]^ If the ankle joint space distance exceeds the range of 3 to 5 mm at the 1-year follow-up under the weight-bearing condition, the prognosis is usually better than if the ankle joint space distance is less than 3 to 5 mm.^[3,21,27,29,42]^ AOS score was another significant indicator of ankle survival as reported by Nguyen et al,^[35]^ who concluded that the patients with a AOS score of less than 42 at 2 years postoperatively have better outcomes. Age as a prognostic indicator after ankle detraction is controversial. It is believed among some scholars^[35]^ that the patients aged over 60 years can obtain better results, while other^[31]^ reports claim that the recovery effect after ADA bears no association with age. Gender is also likely a contributory factor to distraction arthroplasty failure.^[34]^ It was revealed that females suffered a higher rate of treatment failure.^[34]^ In the present study, a man approaching the age of 60 was found to have adequate ankle distraction distance during follow-up, and these factors contributed to a desirable outcome. However, unfortunately, AOS scores at 2 years postoperatively were not evaluated in this study.

With regard to the treatment effect of ADA alone and combined treatment, as recommended by scholars, applying the combined strategies for AA could produce the optimal treatment effect.^[31]^ The common surgical options include arthroscopy, arthrotomy, or superolateral osteotomy combined with ADA.^[4,31]^ Zhang et al^[4]^ suggested that the combination of ankle distraction arthroplasty and arthroscopic microfracture could achieve better results in respect of pain relief and functional recovery for AA than if ADA is used alone. Previous studies show that the clinical outcomes of ankle distraction are better compared to debridement.^[28,43]^

Moreover, this combination therapy has significantly improved the mean AOFAS scores and SF-36 scores. As suggested by these findings, ankle distraction may be more conducive to functional improvement and pain relief when combined with other appropriate approaches. In our study, though only ADA was applied, the AOFAS score, VAS score, SF-36 PCS score, and AAS score improved after operation. Moreover, the results were partially better compared to previous studies.^[44–46]^ According to our analysis, this may be related to the standard surgical techniques, the strict control of indications, the prescription of perioperative ankle motion exercises by the surgeon, and the small number of cases in this study.

As regards the complications ADA, pin-site infection is the most frequent surgical complication of ADA.^[1,3]^ Xu et al^[3]^ reported 2 cases of pin-site infection in their study, which was successfully solved by routinely dressing changes and oral antibiotics. The reported incidence ranges from 14% to 100%.^[28,30,31,33,35,44]^ Osteomyelitis requiring hospitalization and intravenous antibiotics is rare, with a reported incidence ranging from 1.2% to 5.5%.^[28,30,47]^ Besides, K-wire breakage and deep vein thrombosis were also reported in the literature.^[1]^ In most cases, this breakage occurs at the junction of the wire connectors onto the ring. Therefore, it can be corrected by modifying the connection of the needle fixation bolt closer to the skin.^[1]^ The estimated incidence ranges from 14% to 24% in 2 studies of 74 patients.^[28,44]^

It is necessary to educate patients about the correct use of external fixation devices before operation and discharge. Besides, Bernstein et al^[1]^ suggested the use of 50% hydrogen peroxide and 50% normal saline solution and sterile cotton swabs for daily needle position care.

A deep understanding of the anatomy of the lower extremities can avoid accidental damage to the neurovascular structure. In particular, there is a risk of injury to the anterior tibial tendon and the anterior neurovascular bundle during the process of tibial ring installation. To prevent bone thermal injury and needle position infection, surgeons are supposed to use a new, sharp 4.8 mm half needles in each case. To avoid the medial neurovascular structure, great care should be taken when the foot ring is used. Nevertheless, Bernstein et al^[1]^ reported that patients complained of heel numbness, which can be caused by medial calcaneal branch nerve irritation from the crossed hindfoot wires. The symptoms of plantar numbness of forefoot should not be misdiagnosed as tibial nerve injury. The compromise of the posterior tibial nerve is frequent to occur with cute distractions which more than 5 mm.^[1]^ Besides, Tellisi et al^[31]^ claimed that they would not carry out longer than 5 to 6 mm for acute ankle joint distraction in the operating room, and the remaining distraction distance, if demanded, could be performed during the postoperative hospitalization.

In our study, no complications were found during 13 months follow-up period. Although our initial distraction distance was 7 mm, it was slightly larger than the safety distance reported in the literature. However, our surgery is performed under intraoperative neuralogical monitoring. To protect the soft tissue, each set of pins was wrapped in a 2-inch gauze. This positive outcome is attribute to standardized surgical techniques, regular follow-up, intraoperative neuralogical monitoring, and careful perioperative care.

Despite the satisfying clinical outcomes achieved in this case, there remain several limitations. The number of cases is relatively small, so that it is necessary to further conduct a large number of randomized controlled trials. Besides, good clinical results were obtained in the short-term follow-up of this study, and the long-term follow-up results need to be further evaluated.

In conclusion, ADA is reliable to achieve pain relief, functional recovery, and serve AA resolution. Besides, it is an alternative to ankle arthrodesis or total ankle arthroplasty in selected patients with severe AA.

## Author contributions

**Conceptualization:** Bing-Zhe Huang.

**Data curation:** Xiao-Ning Liu, Fei Chang, Zhuan Zhong, Pan Xue.

**Formal analysis:** Xiao-Ning Liu, Fei Chang, Zhuan Zhong.

**Investigation:** Han-Yang Zhang, Bing-Zhe Huang.

**Methodology:** Xiao-Ning Liu, Han-Yang Zhang, Zhuan Zhong, Pan Xue.

**Project administration:** Fei Chang.

**Resources:** Fei Chang, Pan Xue.

**Supervision:** Bing-Zhe Huang.

**Writing – original draft:** Xiao-Ning Liu, Fei Chang.

**Writing – review & editing:** Bing-Zhe Huang.
